# Supplementary material for: Resistance Training in Breast Cancer Survivors: A Systematic Review of Exercise Programs
Source: Int J Environ Res Public Health. 2020 Sep 7;17(18):6511. doi: 10.3390/ijerph17186511 (PMC7558202; doi:10.3390/ijerph17186511)
Supplement: Supplementary file 1 [file ijerph-17-06511-s001.zip › Supplementary Table 1. Characteristics of controlled trials reviewed (1).docx]

**Supplementary Table 1.** Characteristics of controlled trials reviewed.

| **Trial** | **Authors** | **N** | **Age** | **Stage of Cancer** | | **Aim** | **Timing of Exercise** | **Treatment** | **Control Groups** |
| --- | --- | --- | --- | --- | --- | --- | --- | --- | --- |
|  | **During treatment** | | | | | | | | |
| START | Courneya et al., 2007^[45]^  Courneya et al., 2007^[49]^  Courneya et al., 2014^[9]^  Adams et al., 2016^[50]^ | 242 | 49.2 | I–IIIA | | To evaluate the relative merits of AET and RT in blunting these effects. | DC | M-C | AET-UC |
|  |  |  |  |  | | To report a 6-month follow-up of exercise behavior and patient-rated outcomes from an exercise trial in breast cancer patients. |  |  |  |
|  |  |  |  |  | | To report an exploratory follow-up of cancer outcomes from the Supervised Trial of Aerobic versus Resistance Training (START). |  |  |  |
|  | Schwartz et al., 2007^[37]^ | 66 | 50.1±8.7 | I-III | | To test the effects of AER and RT on changes in bone mineral density (BMD) in women newly diagnosed with stage I–III breast cancer receiving chemotherapy. | DC | C-R | AET-UC |
|  | Schwartz & Winters-Stone, 2009^[38]^ | 101 | 47±9.4 | I-III | | To compare differences in weight change and body composition among newly diagnosed cancer survivors receiving chemotherapy. | DC | C | AET-UC |
|  | Sagen et al., 2009^[46]^ | 204 | 55±10 | I-III | | To evaluate the development of ALE in two different rehabilitation programs. | DR/C/HT | M/AND- C-R-HT | UC |
| BEATE | Schmidt et al., 2013^[20]^  Schmidt et al., 2015^[21]^ | 95 | 52.7±10 | I-IV | | Study protocol  To investigate whether RT during chemotherapy provides benefits on fatigue and QoL beyond potential psychosocial effects of group-based interventions. | DC | M/L- C | PMR |
| BEST | Potthoff et al., 2013^[22]^  Steindorf et al., 2014^[8]^  Schmidt et al., 2016^[23]^  Wiskemann et al., 2017^[24]^ | 155 | 55.8±9.1 | 0-III | | Study protocol  To assess the efficacy of 12-week RT on fatigue beyond possible psychosocial effects of a group-based intervention in breast cancer patients during adjuvant radiotherapy. | DR | NC- M/L- R HT | PMR |
|  |  |  |  |  | | To explore the mediating role of inflammatory parameters in the development of fatigue, pain, and potentially related depressive symptoms during radiation therapy for breast cancer and its mitigation by RT. |  |  |  |
|  | **Post treatment** | | | | | | | | |
| WTBS | Schmitz et al., 2005^[47]^  Ohira et al., 2006^[52]^  Ahmed et al., 2006^[10]^ | 79 | 53.3±8.7 | I-III | | To assess the safety and effects of twice-weekly RT among recent BCS. | PT (4-36 months. During hormonal therapy) | AND- C-R | NO |
|  |  |  |  |  | | To examine the effects of weight training on changes in QOL and depressive symptoms in recent BCS. |  |  |  |
|  |  |  |  |  | | To examine effects of supervised upper- and lower-body weight training on the incidence and symptoms of lymphedema in 45 BCS. |  |  |  |
|  | Twiss et al., 2009^[40]^ | 110 | 58.7±7.5 | 0-II | | To determine if 110 postmenopausal BCS had improved muscle strength and balance and had fewer falls compared to BCS who did not exercise; and to describe type and frequency of RT exercises. | PT (>6 months post treatment) | S-C-R | UC |
|  | Musanti, 2012^[42]^ | 42 | 50.5 | I-IIIB | | To compare the effect of combination modality versus single-modality exercise on PSE and GSE and to explore the relationship between exercise modality and the subdomains of PSE. | PT (> 3 months post chemotherapy. > 6 weeks post radiotherapy) | C-R | AET-CT-F |
|  | Schmidt et al., 2012^[35]^ | 33 | 58±8.4 | I-III | | To identify alternative sports intervention in the treatment of BCS. | PT | M/L- C-R | CGE |
|  | Simonavice et al., 2014^[39]^ | 23 | 64±5 | 0-III | | To examine the effects of RT and dried plum consumption on strength, body composition, blood markers of bone, and inflammation in BCS. | PT (≥ 6 months post treatment) | S-C-R-HT | RT+DP |
| Hagstrom and colleagues | Hagstrom et al., 2015^[7]^  Hagstrom et al., 2016^[53]^ | 39 | 51.9±8.8 | I - IIIA | | To evaluate the benefits of resistance training on QoL and fatigue in BCS as an adjunct to UC. | PT (11.6 ± 13.2 months post treatment) | S-C- R-HT | UC |
|  | Hagstrom, A. D., Shorter, K. A., & Marshall, P. W. 2019^[54]^  Hagstrom, A., & Denham, J. 2018^[55]^ |  |  |  | | To determine the effects of RT on markers of inflammation and immune function in BCS. |  |  |  |
| PAL | Schmitz et al., 2009^[25]^  Schmitz et al., 2009^[26]^  Speck et al., 2010^[27]^  Schmitz et al., 2010^[28]^  Hayes et al., 2011^[29]^ Brown et al., 2012^[30]^  Winters-Stone et al., 2014^[31]^  Brown & Schmitz et al., 2015^[32]^  Brown & Schmitz et al., 2015^[33]^  Buchan et al., 2016^[34]^ | 295 | 55.3±8.5 | I-III | | Study protocol  To assess the effects of controlled weight lifting in BCS with lymphedema | NR | S/AND- C. HT | UC |
|  |  |  |  |  | | To evaluate the impact of a twice-weekly RT intervention on perceptions of body image in 234 BCS who participated in the Physical Activity and Lymphedema (PAL) trial. |  |  |  |
|  |  |  |  |  | | To evaluate lymphedema onset after a 1-year weight lifting intervention vs no exercise among survivors at risk for BCRL. |  |  |  |
|  |  |  |  |  | | To compare baseline lymphedema prevalence in the physical activity and lymphedema (PAL) trial cohort and to subsequently compared the effect of the weight-lifting intervention on lymphedema, according to four standard diagnostic methods. |  |  |  |
|  |  |  |  |  | | To promote translation of an efficacious rehabilitative exercise program for BCS by clarifying for clinicians the safety profile of participants. |  |  |  |
|  |  |  |  |  | | To determine whether the Physical Activity and Lymphedema (PAL) trial weight training program for BCS at risk of or with BCRL provided skeletal benefits. |  |  |  |
|  |  |  |  |  | | We explored the potential efficacy of slowly progressive weight lifting to attenuate the decline of ASMM (appendicular skeletal muscle mass) among BCS by conducting a post hoc analysis of data from the Physical Activity and Lymphedema trial. |  |  |  |
|  |  |  |  | |  | To explore the potential efficacy of slowly progressive weight lifting to reduce the incidence of physical function deterioration among BCS |  |  |  |
|  | Cormie et al., 2013^[41]^ | 62 | 57±10 | 0-III | | This study compared the effects of high load and low load resistance exercise on the extent of swelling, severity of symptoms, physical function and quality of life in women with BCRL. | NR | S/AND-C-R-HT | UC |

START= Supervised Trial of Aerobic Versus Resistance Training; BEATE = exercise and relaxation as therapy against fatigue; BEST exercise and relaxation for breast cancer patients during radiotherapy; WTBS= Weight Training for Breast Cancer Survivors; PAL= Physical Activity and Lymphoedema; DC= During chemotherapy; DR= During radiotherapy; DR/C/HT= During radio or chemotherapy, or hormonal therapy; PT= post treatment (surgery, radiotherapy, chemotherapy); NR= not reported; R= radiotherapy; C= chemotherapy; NC= Neoadjuvant chemotherapy; HT= hormonal therapy; S= surgery; M= mastectomy; BM= bilateral mastectomy; L= lumpectomy; AND= axillary node dissection; RT= resistance training; AET= aerobic training; PMR= Progressive muscle relaxation; UC= usual care; CGE= conventional gymnastics exercise; F= Flexibility; CT= combined training; RT+DP= resistance training+dried plum; QoL= quality of life; BCS= breast cancer survivors; BCRL= breast cancer-related lymphedema
