# Supplementary material for: Resistance Training in Breast Cancer Survivors: A Systematic Review of Exercise Programs
Source: Int J Environ Res Public Health. 2020 Sep 7;17(18):6511. doi: 10.3390/ijerph17186511 (PMC7558202; doi:10.3390/ijerph17186511)
Supplement: Supplementary file 1 [file ijerph-17-06511-s001.zip › Supplementary Table 2. Outcomes, measuring tools and main findings. (1).docx]

**Supplementary Table 2.** Outcomes, measuring tools and main findings.

| **Trial** | **Author** | **Outcome measurement** | **Evaluation** | **Results** | |  |
| --- | --- | --- | --- | --- | --- | --- |
|  | **During treatment** | | | | |  |
| START | | Courneya et al., 2007^[45]^              Courneya et al., 2007^[49]^          Courneya et al., 2014^[9]^       Adams et al., 2016^[50]^ | Muscle strength | 8RM | ↑UB and LB strength | |
|  |  |  | Aerobic ﬁtness (V·O2max) | Maximal incremental exercise protocol on a treadmill. | ↑ LBM | |
|  |  |  | Body composition (lean body mass and body fat) | DXA | ↑ QoL | |
|  |  |  | Lymphedema | Standard volumetric (water displacement) | ↓ Fatigue | |
|  |  |  | QoL and fatigue | Functional Evaluation of Cancer Therapy–Anemia (FACT-An) | ↔ Aerobic ﬁtness | |
|  |  |  | Psychosocial functioning | Rosenberg Self-Esteem Scale | ↔ Volume | |
|  |  |  |  | Center for Epidemiological Studies Depression Scale | No adverse events | |
|  |  |  |  | Spielberger State Anxiety Inventory | ↑ Self-esteem | |
|  |  |  | QoL and fatigue | Functional Evaluation of Cancer Therapy–Anemia (FACT-An) |  | |
|  |  |  | Psychosocial functioning | Rosenberg Self-Esteem Scale | ↑ QoL | |
|  |  |  |  | Center for Epidemiological Studies Depression Scale | ↓ Fatigue | |
|  |  |  |  | Spielberger State Anxiety Inventory | ↑ Self-esteem | |
|  |  |  | Exercise during 6-month follow-up | The Godin Leisure Time Exercise Questionnaire | ↓ Anxiety | |
|  |  |  | Follow-up of 89 months | Disease free survival (DFS) | Stronger effects on DFS, OS, DDFS, RFI | |
|  |  |  |  | Overall survival (OS) |  | |
|  |  |  |  | Distant DFS (DDFS), |  | |
|  |  |  |  | Recurrence-free interval (RFI). |  | |
|  | | Schwartz et al., 2007^[37]^ | Muscle strength | 1RM | ↑ Muscle strength | |
|  | |  | BMD (lumbar spine and whole body) | DXA | Attenuates the decrease in BMD | |
|  | |  | Aerobic capacity | 12-minute walk | (↑) Aerobic capacity | |
|  | | Schwartz & Winters-Stone, 2009^[38]^ | Muscle strength | 1RM | ↑ Muscle strength | |
|  | |  | Aerobic capacity | 12-minute walk | ↑ Aerobic capacity (11%) | |
|  | |  | Body composition (body fat) | DXA | ↔ Body composition (body fat) | |
|  | |  | Body weight |  | Maintained body weight | |
|  | |  |  |  | No adverse events | |
|  | | Sagen et al., 2009^[46]^ | Lymphedema (Voldiff (in ml)) | Simplified Water Displacement Instrument (SWDI) | ↔ Volume | |
|  | |  | Pain and the sensation of heaviness in the affected limb during physical activity | Visual analogue scales (VAS) | ↑VAS ratings | |
|  | |  | BMI | Height and weight | Individuals with a baseline BMI >25 kg/m had a ↑ risk of development of ALE at 2 years | |
|  | |  |  |  | No adverse events | |
| BEATE | | Schmidt et al., 2013^[20]^  Schmidt et al., 2015^[21]^ | Fatigue | Fatigue evaluation questionnaire (FAQ) | ↓ Fatigue | |
|  |  |  | QoL | EORTC QLQ-C30 / BR23 | ↑ QoL (role function, social function) | |
|  |  |  | Depression | Center for Epidemiological Studies Depression Scale (CES-D) | ↔ Depression | |
|  |  |  | Cognitive performance | Trail-making-test | (↑) Cognitive performance | |
|  |  |  | Muscle strength | Isometric and isokinetic strength of representative muscle groups for upper and lower extremity measured at the IsoMed2000® | ↑Isometric and isokinetic muscle strength | |
|  |  |  | Cardiorespiratory fitness | Spiroergometry (VO2peak; VO2 at ventilatory threshold) | ↔ Cardiorespiratory ﬁtness (VO2peak) | |
|  |  |  | Safety of training interventions | Lymphedema, pain, nausea, dyspnea, tachycardia, or other disorders assessed by questionnaire | No adverse events | |
| BEST | | Potthoff et al., 2013^[22]^  Steindorf et al., 2014^[8]^ | Fatigue | Fatigue evaluation questionnaire (FAQ) | ↓ Fatigue (total fatigue, physical fatigue) | |
|  |  |  | QoL | EORTC QLQ-C30 / BR23 | ↑ Global QoL (role function, pain); ↑ Emotional function, social function, and body image | |
|  |  |  | Depressive symptoms | Center for Epidemiological Studies Depression Scale (CES-D) | ↔ Depressive symptoms | |
|  |  |  | Cognitive function | Trail-making-test | (↑) Cognitive performance | |
|  |  |  | Muscle strength | Isometric and isokinetic strength of representative muscle groups for upper and lower extremity measured at the IsoMed2000® |  | |
|  |  |  | Endurance performance | Spiroergometry (VO2peak) |  | |
|  |  |  | Safety of training interventions | Number of participants with lymphedema, pain, nausea, dyspnea, or tachycardia during the intervention phase. Using standardized questionnaires. | No adverse events | |
|  |  | Schmidt et al., 2016^[23]^ | Inﬂammatory markers | Analyzed in peripheral blood and urine | ↓ Inﬂammatory markers (IL-6, IL-6/IL-1ra) | |
|  |  |  | Fatigue | Fatigue evaluation questionnaire (FAQ) | ↓ Fatigue | |
|  |  |  | Depressive symptoms | Center for Epidemiological Studies Depression Scale (CES-D) | ↔ Depressive symptoms | |
|  |  | Wiskemann et al., 2017^[24]^ | Pain | European Organization for Research and Treatments of Cancer QLQ-C30 questionnaire | ↓ Pain | |
|  |  |  |  |  | (↑) BMI (BMI is not considered as a mediator of the exercise effect on IL-6) | |
|  |  |  |  |  | No adverse events | |
|  | | **Post treatment** | | | | |
| WTBS | | Schmitz et al., 2005^[47]^ | Muscle strength | 1RM | ↑ Muscle strength (bench press: 63% in immediate versus 12% in delayed treatment groups. The delayed treatment group increased bench press strength 1-RM by 36% from 7 to 12 months) | |
|  |  |  | Lymphedema | Arm-circumference | ↔ Circumference | |
|  |  |  |  | Self-report of diagnosis, and self-report of symptoms |  | |
|  |  |  | Physical activity | Physical Activity Readiness Questionnaire (PAR-Q) | Energy intake and physical activity outside of weight training did not alter the findings | |
|  |  |  |  | The Baecke Questionnaire Physical Activity Readiness Questionnaire (PAR-Q) |  | |
|  |  |  | Energy intake | National Cancer Institute- Diet History Questionnaire (NIH-DHQ) |  | |
|  |  |  | Body composition (lean mass, body fat, bone density) | DXA | ↑Lean mass ↑Body fat % ↔ Body fat | |
|  |  |  | BMI | Height and weight | ↔ Body weight, BMI | |
|  |  |  | Insulin, and insulin-like growth factor axis proteins | Fasting blood glucose and plasma insulin levels | ↓ IGF-II levels | |
|  |  |  |  |  | No adverse events | |
|  |  | Ohira et al., 2006^[52]^ | QoL | Cancer rehabilitation evaluation system short form (CARES-SF) | ↑QoL (Changes in physical global, psychosocial global were correlated with changes in bench press and total lean mass) | |
|  |  |  | Depressive symptoms | Center for Epidemiologic Studies-Depression Scale (CES-D) | ↔ Depressive symptoms | |
|  |  | Ahmed et al., 2006^[10]^ | Lymphedema | Arm-circumference | ↔ Lymphedema/lymphedema symptoms | |
|  |  |  |  | Self-report of diagnosis, and self-report of symptoms |  | |
|  | | Twiss et al., 2009^[40]^ | Muscle strength | Biodex Velocity Spectrum Evaluation | ↑ Muscle strength | |
|  | |  | Dynamic balance | Timed Backward Tandem Walk | ↑ Balance | |
|  | |  | Incidence of Fractures and/or Falls Documentation | Self-report | ↔ Incidence of fractures or falls | |
|  | | Musanti, 2012^[42]^ | Muscular strength | 6RM | ↑ Muscle strength | |
|  | |  | Muscular endurance | Curl-up test |  | |
|  | |  |  | YMCA Bench Press Endurance Test |  | |
|  | |  | Aerobic fitness | Submaximal graded exercise treadmill test (modified Bruce protocol) |  | |
|  | |  | Flexibility (hip and shoulder) | Standard goniometer | (↑) Flexibility | |
|  | |  | Body composition | Bioelectric impedance analysis |  | |
|  | |  | Self-perceptions | Physical Self-perception Profile (PSPP) - Rosenberg Self-Esteem Scale | ↑ PSPP (Physical Self-Esteem: Physical Strength and Attractive body) | |
|  | |  | Fatigue | Piper Fatigue Scale (PFS) | ↓ Fatigue | |
|  | |  | Depression | Hospital Anxiety and Depression Scale (HADS) | ↓ Depressive symptoms | |
|  | | Schmidt et al., 2012^[35]^ | Muscular strength | Hypothetical maximum force test (h1RM) |  | |
|  | |  | Aerobic fitness | Prediction of V·O2max - Bicycle ergometer | (↑) Aerobic fitness | |
|  | |  |  |  | ↑ Perceived exertion | |
|  | |  | QoL | EORTC QLQ-C30 / BR23 | ↑ QoL- fatigue | |
|  | |  | BMI | Height and weight | (↓) BMI | |
|  | | Simonavice et al., 2014^[39]^ | Muscular strength | 1RM | ↑ Muscle strength | |
|  | |  | Body composition (BMD) | DXA | ↔ Body composition | |
|  | |  | BMI | Height and weight |  | |
|  | |  | Biochemical markers of bone formation and inﬂammation | Fasted blood | Maintenance of bone formation, with a decline in bone resorption. | |
| Hagstrom and colleagues | | Hagstrom et al., 2015^[7]^ | Muscular strength | Lower body: 1-RM | ↑ Muscle strength | |
|  |  |  |  | Upper body : unilateral isometric strength |  | |
|  |  |  | QoL | Functional Evaluation of Cancer Therapy – General (FACT-G) Quality of Life Instrument | ↑ QoL | |
|  |  |  | Changes in exercise | Godin Leisure-Time Exercise Questionnaire |  | |
|  |  |  | Fatigue | Functional Evaluation of Cancer Therapy – Fatigue scale (FACIT-fatigue) | ↓ Fatigue | |
|  |  | Hagstrom et al., 2016^[53]^ | Body composition (% body fat - BMI) | Yuhasz formula | ↔ Body composition | |
|  |  | Hagstrom, A. D., Shorter, K. A., & Marshall, P. W. 2019^[54]^  Hagstrom, A., & Denham, J. 2018^[55]^ | Blood markers : Natural killer cell (NK) and natural killer T-cell (NKT) function | Venous blood | ↓ TNF-a on their NK cells | |
|  |  |  | Inﬂammatory markers: TNF-α, IL-6, IL-10, and CRP |  | ↓ TNF-a on their NKT cells | |
|  |  |  |  |  | Correlations between changes in lower body strength and TNF-a expression on NK and NKT | |
| PAL | | Schmitz et al., 2009^[25]^  Schmitz et al., 2009^[26]^ | Lymphedema (swelling, symptoms) | Water displacement method (WDM) | ↔ Lymphedema (swelling, symptoms) | |
|  |  |  |  |  | ↓ Number and severity of symptoms | |
|  |  | Speck et al., 2010^[27]^ | Body image (self-perceptions of appearance, health, physical strength, sexuality, relationships, and social functioning) | Body Image and Relationships Scale (BIRS) | ↑ BIRS Total, strength and health, appearance and sexuality | |
|  |  |  | QoL | SF-36 | ↑ SF-36 (mental composite) | |
|  |  | Schmitz et al., 2010^[28]^ | Body composition (BMD, body fat) | DXA | ↔ Body composition | |
|  |  |  | Lymphedema (swelling, symptoms) | Water displacement method (WDM) | ↔ Lymphedema (swelling, symptoms) | |
|  |  | Hayes et al., 2011^[29]^ | Lymphedema | Volumetric: Water displacement method | ↔ Interlimb volume | |
|  |  |  |  | Sum of arm circumferences | ↔ Interlimb sum of circumference | |
|  |  |  |  | Bioimpedance spectroscopy | ↔ Interlimb ratio | |
|  |  |  |  | Norman lymphedema survey | ↔ Norman score | |
|  |  |  |  |  | ↔ among the four standard diagnostic methods | |
|  |  | Brown et al., 2012^[30]^ | Body composition (BMD, body fat) | DXA | ↓ Body fat (BCS at risk for lymphedema) | |
|  |  |  | Musculoskeletal Injury | Self-reported musculoskeletal injury | BCS with lymphedema had a greater odds of experiencing a musculoskeletal injury | |
|  |  |  | Health care use | Questionnaire | BCS at risk for lymphedema did not have a higher risk for musculoskeletal injury | |
|  |  | Winters-Stone et al., 2014^[31]^ | Body composition (BMD) | DXA | ↔ T-Score (Lumbar Spine) | |
|  |  | Brown & Schmitz et al., 2015^[32]^ | Appendicular skeletal muscle mass (ASMM) | DXA | Attenuates the muscle mass decline | |
|  |  |  | Body composition (body fat%, fat mass, ) | DXA | ↔ Body composition (body fat %, fat mass, BMI, total body mass) | |
|  |  | Brown & Schmitz et al., 2015^[33]^ | Physical function | SF-36 (physical function subscale) | ↓ Deterioration of physical function | |
|  |  |  |  |  |  | |
|  |  | Buchan et al., 2016^[34]^ | Muscular strength | 1RM/ Maximal grip strength | ↑ Muscle strength (1RM- handgrip) | |
|  |  |  | Physical activity | International Physical Activity Questionnaire | ↔ Diet, physical activity and anthropometric measures | |
|  |  |  | BMI | Height and weight |  | |
|  |  |  | Caloric intake | Diet History Questionnaire |  | |
|  |  |  |  |  | No adverse events | |
|  | Cormie et al., 2013^[41]^ | Lymphedema (Extent of swelling ) | Bioimpedance spectroscopy (BIS) | ↔ BIS, DXA or arm-circumference | |  |
|  |  |  | DXA |  | |  |
|  |  |  | Arm-circumference |  | |  |
|  |  | Lymphedema (Symptom severity) | The disability of the arm, shoulder and hand questionnaire (DASH) | ↔ DASH, BPI, FACT-B+4 or QLQ-BR23 | |  |
|  |  |  | Brief Pain Inventory (BPI). |  | |  |
|  |  |  | The arm morbidity sub-scale of the Functional Evaluation of Chronic Illness Therapy breast cancer questionnaire for patients with lymphedema (FACT-B+4) |  | |  |
|  |  |  | The arm symptoms sub-scale of the European Organization for Research and Treatment of Cancer breast cancer module (QLQ-BR23) |  | |  |
|  |  | QoL | Medical Outcomes Study short-form (SF-36) | ↑ QoL (physical functioning) in the low-load resistance exercise group compared with control | |  |
|  |  | Physical function: |  |  | |  |
|  |  | Muscular strength | Maximal grip strength | (↑) Maximal grip strength | |  |
|  |  |  | 1RM | ↑ Muscle strength | |  |
|  |  | Muscle endurance | Repetition maximum test: maximal number of repetitions possible with 70 % of current 1RM | ↑ Upper body muscle endurance | |  |
|  |  | Range of motion | Standard goniometric techniques | ↑ Shoulder flexion ROM in the low-load resistance exercise group compared with control | |  |
|  |  |  |  | Change in the physical functioning domain was associated with the change in muscle strength | |  |
|  |  |  |  | No adverse events | |  |
| START= Supervised Trial of Aerobic Versus Resistance Training; BEATE = exercise and relaxation as therapy against fatigue; BEST exercise and relaxation for breast cancer patients during radiotherapy; WTBS= Weight Training for Breast Cancer Survivors; PAL= Physical Activity and Lymphoedema; BMI= body mass index; BMD= bone mineral density; V·O2max= Peak oxygen; DXA= dual x-ray absorptiometry; QoL= quality of life; 1RM= one-repetition maximum;8RM= eight repetition maximum; UB= upper body; LB= lower body; LBM= lean body mass; ↑ significant increase; ↓ significant decrease; ↔ without changes; (↑) no significant increase | | | | |  |  |
