# Supplementary material for: Resistance Training in Breast Cancer Survivors: A Systematic Review of Exercise Programs
Source: Int J Environ Res Public Health. 2020 Sep 7;17(18):6511. doi: 10.3390/ijerph17186511 (PMC7558202; doi:10.3390/ijerph17186511)
Supplement: Supplementary file 1 [file ijerph-17-06511-s001.zip › Supplementary Table 3. Exercise prescription. (1).docx]

**Supplementary Table 3.** Exercise prescription

| **Trial** | **Authors** | **Muscular strength Evaluation/Exercise** | **Supervised training** | **Resistance** | **Trial duration (weeks)** | **Exercise intensity, sets, repetitions** | **Sessions/week** | **Session duration/min** | **Muscular groups/training exercises** |  |
| --- | --- | --- | --- | --- | --- | --- | --- | --- | --- | --- |
|  | **During treatment** | | | | | | | | | |
| START | \| Courneya et al., 2007^[45]^  Courneya et al., 2007^[49]^  Courneya et al., 2014^[9]^  Adams et al., 2016^[50]^ \| \| --- \| \|  \| | 8RM/  UB: bench press  LB: leg extension | Yes | NR | Duration of C (median: 17 weeks) | Sets: 2  Reps: 8-12  Intensity: 60-70% estimated 1RM  Increase: 10% (when participants completed >12 reps/set) | 3 | NR | UB: chest press, bicep curl, triceps extension, seated row  LB: knee extension, leg press, leg curl, calf raises |  |
|  | Schwartz et al., 2007^[37]^ | 1RM/  UB: shoulder press, seated row  LB: knee extension | No | Bands | 24 | Sets: 2  Reps: 8-10  Intensity: NR  Increase: modifying starting grip position on resistance bands (when participant completed 2 sets/10 reps) | 4 | NR | NR |  |
|  |  |  |  |  |  |  |  |  |  |  |
|  |  |  |  |  |  |  |  |  |  |  |
|  |  |  |  |  |  |  |  |  |  |  |
|  | Schwartz & Winters-Stone, 2009^[38]^ | 1RM/  UB: seated row, shoulders overhead press  LB: leg press | No | Body weight - Dumbbells - Bands | 48 | Sets: 3/2  Reps: 12/18-20  Intensity: NR  Increase: NR | 4 | 20-30 min | NR |  |
|  |  |  |  |  |  |  |  |  |  |  |
|  |  |  |  |  |  |  |  |  |  |  |
|  |  |  |  |  |  |  |  |  |  |  |
|  | Sagen et al., 2009^[46]^ | NR | Yes | NR | 24 | Sets: NR  Reps: 11-15  Intensity: 0.5 kg for 2 wk  Increase: according to tolerance/symptoms | 3 | 45 min | NR |  |
|  |  |  |  |  |  |  |  |  |  |  |
|  |  |  |  |  |  |  |  |  |  |  |
|  |  |  |  |  |  |  |  |  |  |  |
| BEST / BEATE | Schmidt et al., 2013^[20]^  Schmidt et al., 2015^[21]^  Potthoff et al., 2013^[22]^  Steindorf et al., 2014^[8]^  Schmidt et al., 2016^[23]^  Wiskemann et al., 2017^[24]^ | Ism-Isk /  UB -LB : exercise NR | Yes | Machines | 12 | Sets: 3  Reps: 8-12  Intensity: 60-80% 1RM  Increase: NR | 2 | 60 min | UB: latissimus pull down, shoulder internal and external rotation, shoulder flexion and extension, butterfly and butterfly reverse, seated row  LB: knee extension, leg press, leg curl |  |
|  | **Post treatment** | | | | | | | | | |
| WTBS | Schmitz et al., 2005^[47]^  Ohira et al., 2006^[52]^  Ahmed et al., 2006^[10]^ | 1RM/  UB: bench press  LB: leg press | Yes (12 weeks) | Machines - Body weight - Dumbbells | 48  24 | Sets: 1/3  Reps: 8-10/10-12  Intensity: UB= according to tolerance/symptoms - LB= 8-10RM  Increase: according to tolerance/symptoms | 2 | 60 min | UB: chest, back, shoulders  LB: buttocks, thighs, and legs |  |
|  | Twiss et al., 2009^[40]^ | Isk/  UB: peak torque at 60-degrees in wrist flexion/extension, Nm  LB: peak torque at 60-degrees in hip flexion/extension, knee flexion/extension, Nm.* | No (32 weeks)/ Yes (64 weeks) | Machines - Body weight - Dumbbells | 96 | Sets: 2  Reps: 8-12  Intensity: NR  Increase: according to tolerance/symptoms | 2 | 30-45 min | UB: latissimus pull down, bicep curl, triceps extension, wrist curl, wrist extension, ball-gripping, upward row, push-ups/push-ups on knees, back extension, sit ups  LB: knee extension, leg press, leg curl, lunge, hip flexion- hip extension, side hip raise, calf raises |  |
|  | Musanti, 2012^[42]^ | 6RM/ UB: chest press, seated row - LB: leg press | No | Bands | 12 | Sets: 1  Reps: 10-12  Intensity: RPE of 3–5 on a scale of 0–10  Increase: When their RPE fell to three or lower. | 3 | NR | UB: latissimus pull down, chest press, shoulder press, shoulder flexion and extension, bicep curl, triceps extension, seated row, sit ups  LB: leg press, squat, hip flexion- hip extension |  |
|  | Schmidt et al., 2012^[35]^ | Estimated 1RM/  UB: chest press  LB: leg press | Yes | Machines | 24 | Sets: 2  Reps: 8--12  Intensity: 50% estimated 1RM  Increase: according to tolerance | 1 | NR | UB: latissimus pull down, chest press, shoulder press, bicep curl, triceps extension, seated row, sit ups  LB: leg press, squat, hip flexion- hip extension |  |
|  | Simonavice et al., 2014^[39]^ | 1RM/  UB: chest press  LB: leg press | Yes | Machines | 24 | Sets: 2  Reps: 8-12  Intensity: 60-80% 1RM  Increase: 10% (when participants completed >12 reps/set) | 2 | NR | UB: chest press, bicep curl, triceps extension, triceps pushdown, seated row, lower back hyperextension, sit ups  LB: knee extension, leg press, leg curl |  |
| Hagstrom and colleagues | Hagstrom et al., 2015^[7]^  Hagstrom et al., 2016^[53]^  Hagstrom, A. D., Shorter, K. A., & Marshall, P. W. 2019^[54]^  Hagstrom, A., & Denham, J. 2018^[55]^ | UIsm /  UB: chest press  1-RM/  LB: leg press | Yes | Machines - Body weight - Dumbbells | 16 | Sets: 3  Reps: 8-12  Intensity: 80% 1RM  Increase: NR | 3 | 60 min | UB: latissimus pull down, bench press, one-arm row- barbell bent, seated rowing, back extension, lower back hyperextensions, sit ups  LB: knee extension, leg press, leg curl - prone hold |  |
| PAL | Schmitz et al., 2009^[25]^  Schmitz et al., 2009^[26]^  Speck et al., 2010^[27]^  Schmitz et al., 2010^[28]^  Hayes et al., 2011^[29]^ Brown et al., 2012^[30]^  Winters-Stone et al., 2014^[31]^  Brown & Schmitz et al., 2015^[32]^  Brown & Schmitz et al., 2015^[33]^  Buchan et al., 2016^[34]^ | 1RM/  UB: bench press  LB: leg press | Yes (12 weeks) | Machines - Body weight - Dumbbells | 48 | Sets: 2-3  Reps: 10-12  Intensity: UP: weight or one pound weights for each exercise - LB: 8-10RM  Increase: UB= 1/2 pound after two sessions (according to tolerance/symptoms) - LB= smallest possible increment (when participants completed >10-12 reps/set) | 2 | 60-90 min | UB: chest press, one-arm row- barbell bent, lateral, front and up raise shoulder, bicep curl, triceps pushdown, seated row, back extension  LB: knee extension, leg press, leg curl |  |
|  | Cormie et al., 2013^[41]^ | 1RM/  UB: chest press, seated row  LB: leg press | Yes | NR | 12 | Sets: 1/4  Reps: LL= 20-15RM - HL= 10-6RM  Intensity: LL= 55-65% of 1RM - HL=75-85% of 1RM  Increase: 5-10% after two sessions (when participants completed more than the repetitions specified) | 2 | 60 min | UB: latissimus pull down, chest press, shoulder press, lateral, front and up raise shoulder, bicep curl, triceps extension, wrist curl, seated row  LB: knee extension, leg press, squat, lunge |  |
| \| START= Supervised Trial of Aerobic Versus Resistance Training; BEATE = exercise and relaxation as therapy against fatigue; BEST exercise and relaxation for breast cancer patients during radiotherapy; WTBS= Weight Training for Breast Cancer Survivors; PAL= Physical Activity and Lymphoedema; 8RM= eight repetition maximum; 6RM= eight repetition maximum; 1RM= one-repetition maximum; UB= Upper body strength; LB= Lower body strength; NR= not reported; Repetitions= Reps; HL= high load; LL= low load; C= chemotherapy; Ism-Isk: Isometric and isokinetic strength; Isk: Isokinetic strength; Ism: Isometric strength; UIsm: Unilateral isometric strength. \| \| --- \| \| *Cheema et al., 2014 \| | | | | | | | | | | |
